# Down‐expression of Foxj1 on airway epithelium with impaired cilia architecture in non‐cystic fibrosis bronchiectasis implies disease severity

**DOI:** 10.1111/crj.13605

**Published:** 2023-03-16

**Authors:** Xiao‐Ling Zou, Hai‐Ling Yang, Wen‐Wen Ding, Hai‐Ke Li, Yu‐Qi Zhou, Tian‐Tuo Zhang

**Affiliations:** ^1^ Department of Pulmonary and Critical Care Medicine The Third Affiliated Hospital of Sun Yat‐sen University, Institute of Respiratory Diseases of Sun Yat‐Sen University Guangzhou China

**Keywords:** airway epithelium, cilia disorder, forkhead box protein j1, non‐cystic fibrosis bronchiectasis, pathogenesis

## Abstract

**Introduction:**

The pathogenesis of non‐cystic fibrosis bronchiectasis has not been clearly clarified. This study aimed to investigate the expression of ciliary regulating protein forkhead box protein j1 (Foxj1) on airway epithelium in non‐cystic fibrosis bronchiectasis and its association with airway cilia structure disorder and disease severity.

**Methods:**

Lung tissue sections excised from 47 patients with non‐cystic fibrosis bronchiectasis were included between January 2018 and June 2021. Specimens from 26 subjects who underwent a lobectomy due to lung nodule were chosen as controls. Clinical information was collected, and pathologic analysis was performed to assess the epithelial structure and expression of ciliary regulating Foxj1.

**Results:**

Of the 47 patients with non‐cystic fibrosis bronchiectasis, 25 were considered as mild, 12 were moderate whereas the remaining 10 cases were severe according to the bronchiectasis severity index score evaluation. Epithelial hyperplasia, hyperplasia of goblet cells and inflammatory cell infiltration were observed in non‐cystic fibrosis bronchiectasis, compared with control subjects. Cilia length in non‐cystic fibrosis bronchiectasis patients were shorter than that in the control group, (5.34 ± 0.89) μm versus (7.34 ± 0.71) μm, respectively (*P* = 0.002). The expression of Foxj1 was (2.69 ± 1.09) × 10^6^ in non‐cystic fibrosis bronchiectasis, compared with (6.67 ± 1.15) × 10^6^ in the control group (*P* = 0.001). Moreover, patients with lower expression of Foxj1 showed shorter airway cilia and worse in disease severity.

**Conclusion:**

Foxj1 declined in the airway epithelium of patients with non‐cystic fibrosis bronchiectasis, positively correlated to cilia length and might imply worse disease severity.

## Graphical abstract

The present study found that expression of forkhead box protein j1 (Foxj1) declined in the airway epithelium of patients with non‐CF BE and was related to cilia length and disease severity. It indicated that downregulation of Foxj1 might play an important role in pathogenesis and development of non‐CF BE by modulating airway cilia function.

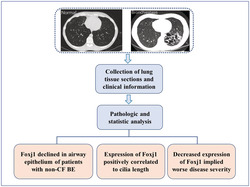

## 1 INTRODUCTION

Non‐cystic fibrosis bronchiectasis (non‐CF BE) is a chronic inflammatory lung disease characterized by permanent abnormal dilatation of the bronchi,^1^ usually accompanied with impaired host defence and recurrent bronchial infection.^2^, ^3^ The prevalence of non‐CF BE is highest in Asian populations,^4^ and the disease has caused significant burden on patients.^5^ The cause of non‐CF BE is multifactorial, and Cole's vicious cycle model is generally accepted for explanation on the occurrence and evolution of the disease.^6^ According to this hypothesis, central to the pathogenesis of non‐CF BE is a cycle of failed bacterial clearance, airway inflammation and airway structural damage.

Motile multi‐ciliated cells, the major cell population in airway epithelium (accounts for 50% to 90%), keep removing inhaled harmful things from the airways in a cephalad direction.^4^, ^7^ Multiple reports have demonstrated that cilia disorder or cilia loss on epithelium of non‐CF BE can lead to impaired clearance activity of cilia.^8^ In non‐CF BE, the airway clearance activity is abnormally slower than that in normal subjects, resulting in sputum retention, and mucous plugs over time, which will further cause airway obstruction, obliteration and damage that lead to more severe bronchiectasis.^9^, ^10^ Stem/progenitor cells, the origin of airway multiciliated cells within basal cell population, are capable to replicate and differentiate into secretory, intermediate and ciliated cells.^11^, ^12^, ^13^ Forkhead box protein j1 (Foxj1) has been demonstrated to be a key regulator of this process^14^, ^15^ and one of the most well characterized transcription factors participating in motile ciliated cell differentiation.^16^, ^17^ Studies have indicated that it also involved in multiple ciliogenesis processes such as centrosome multiplication, docking, cilia elongation and motile cilia formation.^18^ Besides promoting cilia length, high expression of Foxj1 can also induce cilia number increase; whereas, low expression of Foxj1 modulated by inflammatory factors (e.g., IL‐13) may cause cilia architecture impairment or cilia loss in airway epithelium.^19^, ^20^ In another study, researchers found patients with nasal polyps showed increased cilia number per ciliated cell and abnormally lengthened cilia structures, with overexpression of Foxj1 that positively correlated to cilia length compared with healthy control subjects.^13^

Nevertheless, there was quite few researches on the expression of Foxj1 in lower airway epithelium of non‐CF BE and its association with airway cilia disorder and disease severity. Thus, the present study aimed to investigate morphological pattern of motile cilia and Foxj1 expression level on airway epithelium in non‐CF BE, to determine its effect on airway cilia disorder and disease severity, with the purpose to better understand the pathogenesis of non‐CF BE.

## 2 MATERIAL AND METHODS

### 2.1 Study design and patients

Lung tissues excised from 47 non‐CF BE patients who underwent lobectomy for recurrent pulmonary infection or massive hemoptysis resistant to medicine therapy were collected between January 2018 and June 2021 at the Third Affiliated Hospital of Sun Yat‐sen University and the Third Affiliated Hospital of Sun Yat‐sen University, Lingnan Hospital. Non‐CF BE was defined as presence of abnormal dilated bronchial on high‐resolution computerized tomography (CT) scanning accompanied with daily cough with sputum production and recurrent pulmonary infections. All the patients do not have other major pulmonary diagnoses. Exclusion criteria were as follows: cystic fibrosis, allergic bronchopulmonary aspergillosis, interstitial lung disease, active pulmonary tuberculosis, malignant tumour, current smoking within 2 years, HIV infection, hepatic or renal failure or pregnancy. A sweat test has not been performed to exclude cystic fibrosis because of the known rarity of cystic fibrosis among the Chinese and the lack of suggestion of multi‐system disease in any of the patients. Lung function test was performed for each patient. Sputum samples were collected over a maximum of 4 h in sterile pots. Specimens from 26 people who underwent a lobectomy due to lung nodule were chosen as controls, which were proved to be benign by tissue pathology with normal routine blood analyses and normal baseline spirometric values. The control tissue specimens were resected clearly far enough from the lung nodule. This study was conducted respecting the Declaration of Helsinki of the World Medical Association 1964 (and subsequent ratifications) on ethical principles for medical research in humans and approved by the Research Ethics Board of the Third‐Affiliated Hospital (approval number: [2018]02‐158‐01). All participants provided written informed consent.

Clinical information of all the patients enrolled were collected, including gender, age, body mass index (BMI), forced expiratory volume in 1 s (FEV1)% predicted, sputum culture results and exacerbation frequency in the past 1 year. Bronchiectasis severity index (BSI) was calculated for all the non‐CF BE patients. According to the BSI scores, patients were classified into three groups: mild group (low BSI score, overall score 0–4 points), moderate group (intermediate BSI score, overall score 5–8 points) and severe group (high BSI score, overall score 9 or more points).^21^

## 3 MATERIALS AND METHODS

### 3.1 Histopathology

Lung tissues were fixed in formalin, embedded in paraffin, cut in 5 μm sections and stained with haematoxylin and eosin (H&E). Goblet cells were assessed with periodic acid–Schiff (PAS) staining. Stained slides were examined under a light microscope. Pathological alterations, including inflammatory cell infiltration, epithelial and goblet cell hyperplasia were evaluated and recorded. Inflammatory cell infiltration was graded as follows^22^: no cellular infiltration (score 0), few peri‐bronchiolar inflammation (score 1), one layer of peri‐bronchiolar cellular inflammation (score 2), two to four layers of peri‐bronchiolar cellular inflammation (score 3), more than four layers of peri‐bronchiolar cellular inflammation (score 4). Epithelial hyperplasia was graded as follows: one layer (score 0), two to three layers (score 1) and four to six layers (score 3). Goblet cell hyperplasia was evaluated using the following criteria: no goblet cell observed (score 0), one layer (score 1), two layers (score 2) and three layers (score 3). Goblet cell hypertrophy was defined as double to the normal volume.^23^

### 3.2 Immunohistochemical (IHC) staining

Biopsies were embedded in paraffin and sectioned, according to the methods applied in the previous study.^24^ The section was deparaffinized and rehydrated first, then blocked with 0.3% H_2_ O_2_ and heated in citrate buffer at 121°C for 20 min. After staining, the slides were incubated at 4°C overnight; then, rabbit anti‐human dynein heavy chain axonemal polyclonal antibody (DNAH5, Abcam, Cambridge, MA) was used to stain the cilia structure. Afterwards, the slides were incubated at room temperature with Dako Envision +System‐HRP for about 30 min, followed by diaminobenzidine to visualize reaction product and counterstained by haematoxylin. Cilia length was evaluated according to the methods described in previous study.^13^ First, five areas of the stained cilia structure were randomly selected from each slide, and then, cilia length was measured by Image J software. The average cilia length was calculated through 20 measurements for each slide.

### 3.3 Immunofluorescence (IF) staining

IF staining was adopted to assess Foxj1 expression in airway epithelium, and rabbit anti‐human Foxj1 (Proteintech group, Chicago, USA) was used. The procedures were performed as the literature previously described.^25^, ^26^ Tissues were inflated and embedded with Tissue‐Tek OCT medium and cryosectioned at 5 μm, then fixed and permeated with ice‐cold acetone for 20 min and blocked with 10% normal goat serum for 60 min at room temperature. Tissue sections were incubated with primary antibody, followed by Alexa Fluor 488 conjugated secondary antibody (goat anti‐mouse Ab, H + L; MolecularProbes, Carlsbad, CA) at room temperature without light for 60 min. All incubations were performed with 3% normal goat serum, and finally, nuclei were counterstained with (4′,6‐diamidino‐2‐phenylindole) DAPI (0.5 g/mL, Molecular Probes) in phosphate‐buffered saline (PBS). Cilia length was evaluated by assessing type IV‐β tubulin staining. Images were captured with a Zeiss Axiovert 200M fluorescence microscope. Image J software was used to measure Foxj1 expression level by calculating positively stained area value and mean fluorescence intensity (MFI). Finally total fluorescence intensity (TFI) was obtained by multiplying the positive areas with MFI and corrected by subtracting the background autofluorescence.^13^

### 3.4 Statistical analysis

Statistical analysis was performed using SPSS Statistics version 20. Results were expressed as mean ± standard deviation when in accordance with normal distribution and presented as median (interquartile range) if not. Categorical variables were expressed by percentage. Student's *t* test was used to compare differences between continuous variables, whereas Fisher's exact test or Pearson chi‐square test was adopted to compare categorical variables and percentage. Correlation analysis was performed with Spearman r. *P* value < 0.05 was considered statistically significant.

## 4 RESULTS

### 4.1 Demographic data and clinical characteristics

Clinical characteristics were illustrated in Table 1. The average age of the 47 patients with non‐CF BE was (49.17 ± 13.36) years, and male accounted for 46.8% (22 cases). The mean age of the 26 control subjects was (54.7 ± 11.55) years, with 57.6% male (15 cases). There were no significant differences on age and gender between the two groups. Fourteen cases of the 47 non‐CF BE patients showed microbiology positive. *Pseudomonas aeruginosa* (PA) was found in four cases, whereas the other 10 cases showed other bacteria positive. Twenty‐five patients were considered as mild, 12 cases were moderate, and the rest 10 cases were severe according to the BSI scores, with an average of (5.08 ± 4.87). Moreover, the FEV1% predicted was (73.76 ± 24.43)% on average in patients with non‐CF BE.

**TABLE 1:** Clinical characteristics of patients with non‐CF BE.

	Non‐CF BE (*N* = 47)	Value
PA positive	4	8.5%
Other bacteria positive	10	21.3%
FEV1% predicted	73.76 ± 24.43	
Numbers of lung lobe involved		
1	20	42.6%
2	16	34.0%
3	10	21.3%
4	1	2.1%
BSI score	5.08 ± 4.87	
Mild(0–4)	25	53.2%
Moderate(5–8)	12	25.6%
Severe(≥9)	10	21.3%

Abbreviations: BSI, Bronchiectasis Severity Index; FEV1, forced expiratory volume in 1 s; non‐CF BE, non‐cystic fibrosis bronchiectasis; PA, *P. aeruginosa*.

### 4.2 Histopathological lesions in patients with non‐CF BE

**FIGURE 1:**
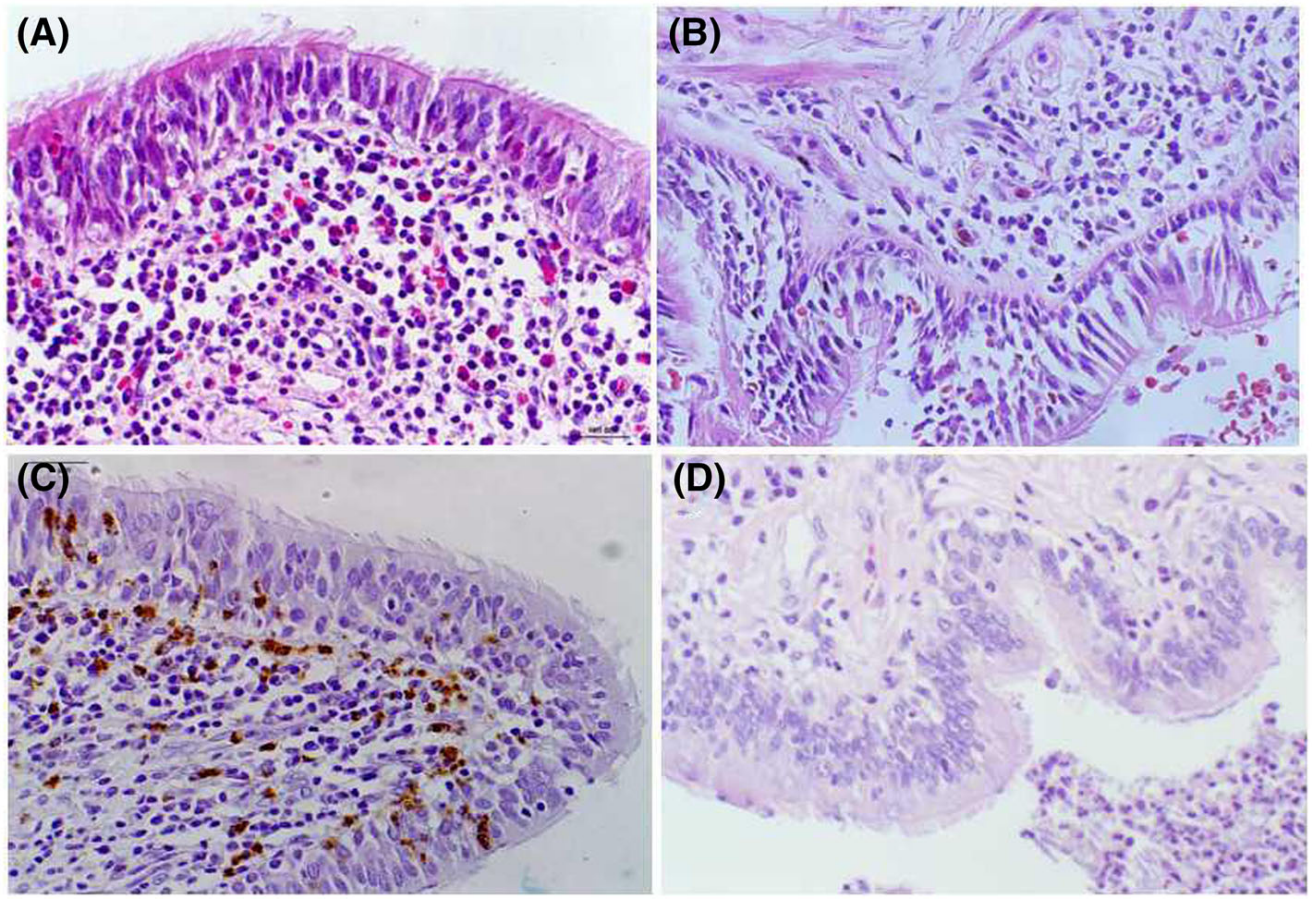
Inflammatory cell infiltration on airway epithelium (×400 magnification). (A) Neutrophilic cell and (C) eosinophilic cell infiltration on airway epithelium of non‐cystic fibrosis bronchiectasis (non‐CF BE); (B) neutrophilic cell and (D) eosinophilic cell staining in control subjects.

**FIGURE 2:**
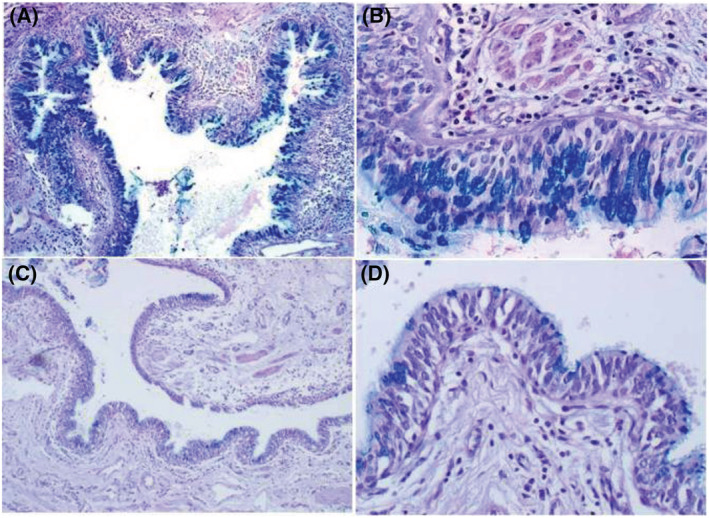
Comparison of airway goblet cell hyperplasia in non‐cystic fibrosis bronchiectasis (non‐CF BE) (A, ×100 magnification; B, ×400 magnification) and control subjects (C, ×100 magnification; D, ×400 magnification).

**FIGURE 3:**
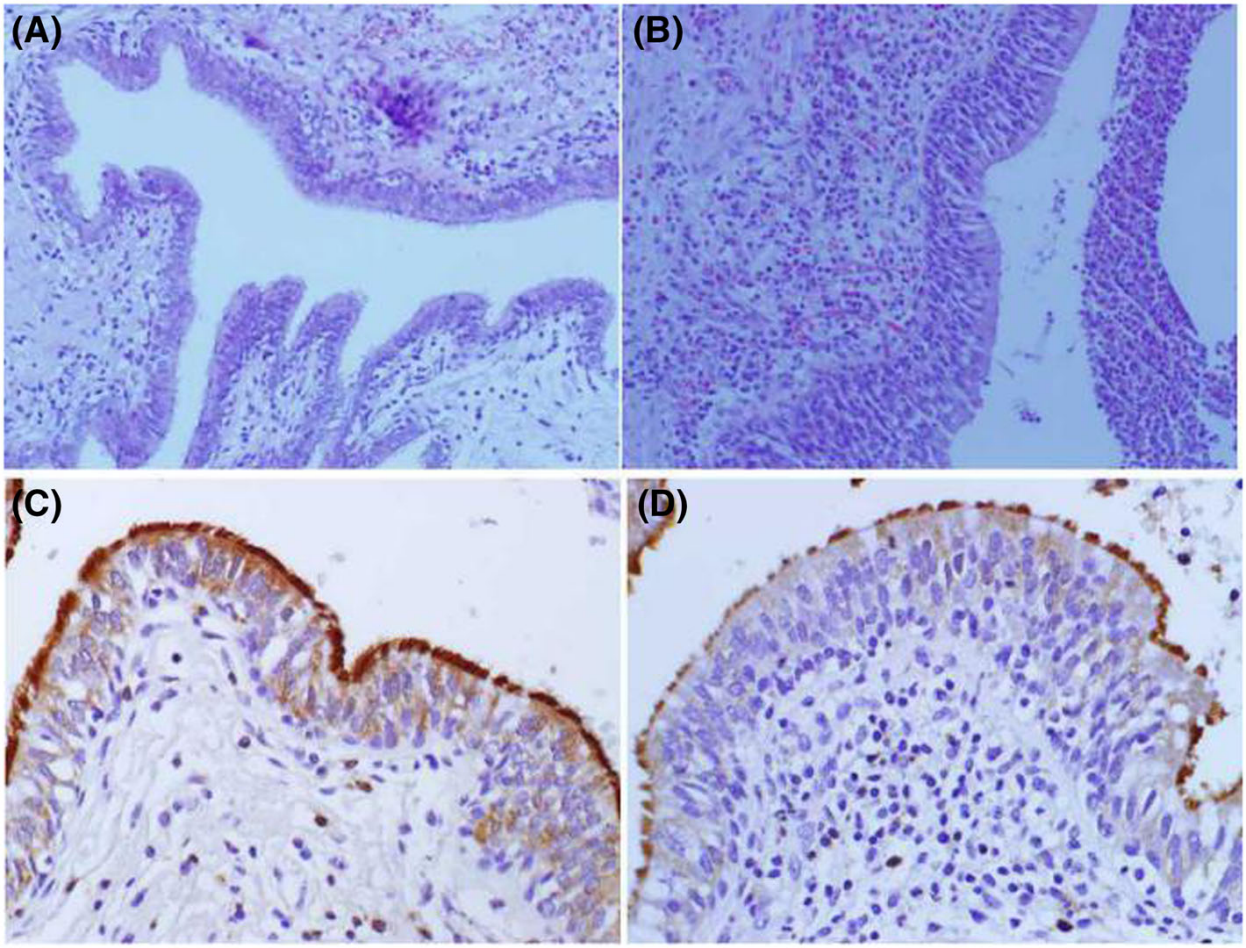
Comparison of airway epithelial hyperplasia (×200 magnification; A, control subjects; B, non‐cystic fibrosis bronchiectasis [non‐CF BE]) and airway cilia architecture (×400 magnification; C, control subjects; D, non‐CF BE).

The bronchial and alveolar structures of control subjects were normal and regular, with few peribronchial and perivascular inflammation and rare PAS positive cell in the airway. Compared with the control group, abnormal dilation of bronchus, epithelial hyperplasia and increased inflammatory cell infiltration (including eosinophilic, lymphocytic and neutrophilic inflammation) were found in patients with non‐CF BE (shown in Figures 1, 2, 3). Moreover, PAS staining results indicated that hyperplasia and hypertrophy of goblet cell were also observed in airway of these patients. When evaluating the extent of airway epithelial hyperplasia, inflammatory cell infiltration and goblet hyperplasia, patients from non‐CF BE group got higher scores than the control group. The differences between the two groups were statistically significant (Table 2).

**TABLE 2:** Comparison of histopathological lesions in non‐CF BE and control subjects.

	Non‐CF BE group	Control group	*P*
Inflammatory cell infiltration	2.46 ± 0.69	0.61 ± 0.57	0.005
Epithelial hyperplasia	2.38 ± 0.49	0.38 ± 0.29	0.012
Goblet cell hyperplasia	2.32 ± 0.75	0.51 ± 0.47	0.015

Abbreviation: Non‐CF BE, non‐cystic fibrosis bronchiectasis.

### 4.3 Airway cilia impairment in patients with non‐CF BE

**FIGURE 4:**
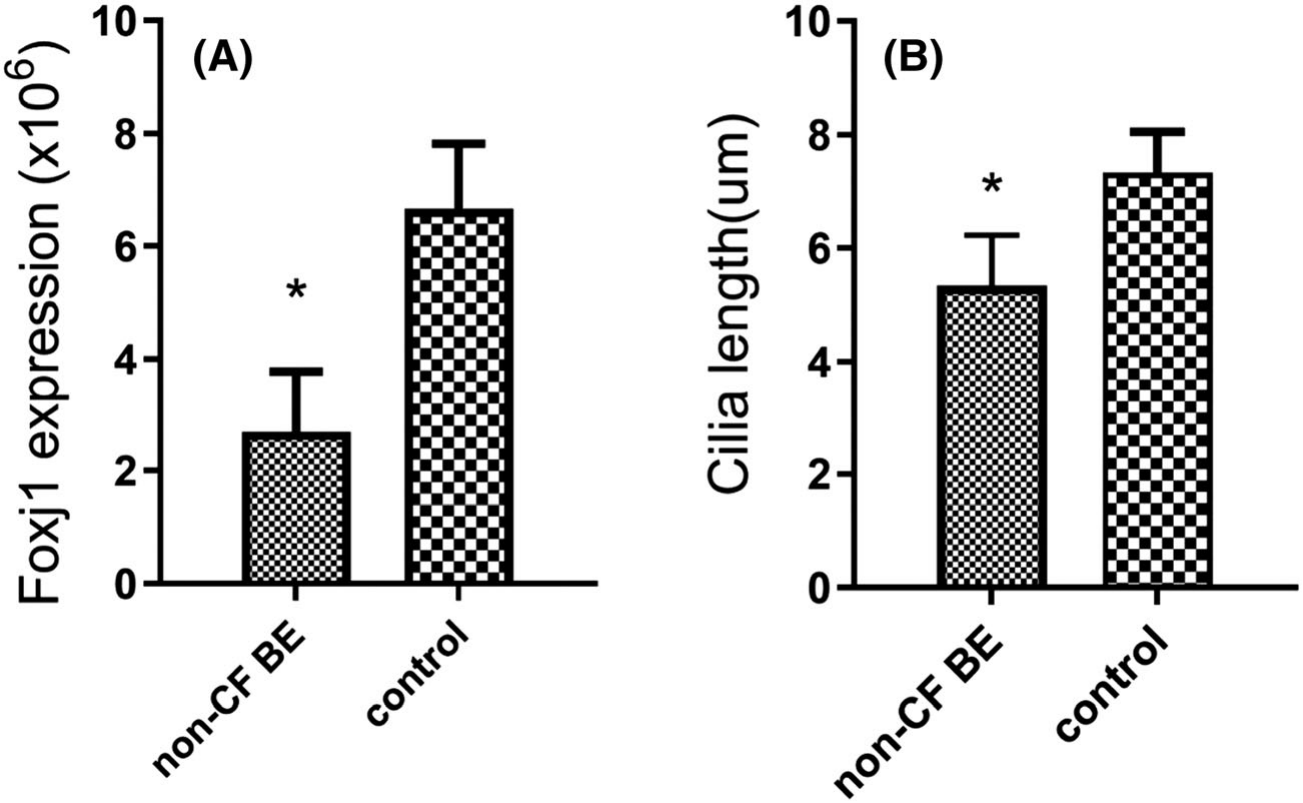
Comparison of cilia length (A, *P = 0.002 versus control group) and forkhead box protein j1 (Foxj1) expression (B, *P = 0.001 versus control group) in non‐cystic fibrosis bronchiectasis (non‐CF BE) group and control group.

Cilia morphology was evaluated after staining with DNAH5. In healthy control subjects, the cilia architectures were normal and regular as expected. By contrast, in the airways of non‐CF BE patients, abnormal cilia architectures with untidy, irregular arrangement and unequal length were observed on the epithelial surface. For most of the patients, shortening of cilia or absence of cilia architecture were observed on the airway epithelium, whereasfew patients showed abnormally dense and lengthened cilia structures (Figure 3). The median cilia length in non‐CF BE patients was shorter than that in the control group, (5.34 ± 0.89) μm versus (7.34 ± 0.71) μm, respectively (Figure 4, *P* = 0.002).

### 4.4 Expression of Foxj1 in airway of non‐CF BE patients and its association with cilia length and disease severity

**FIGURE 5:**
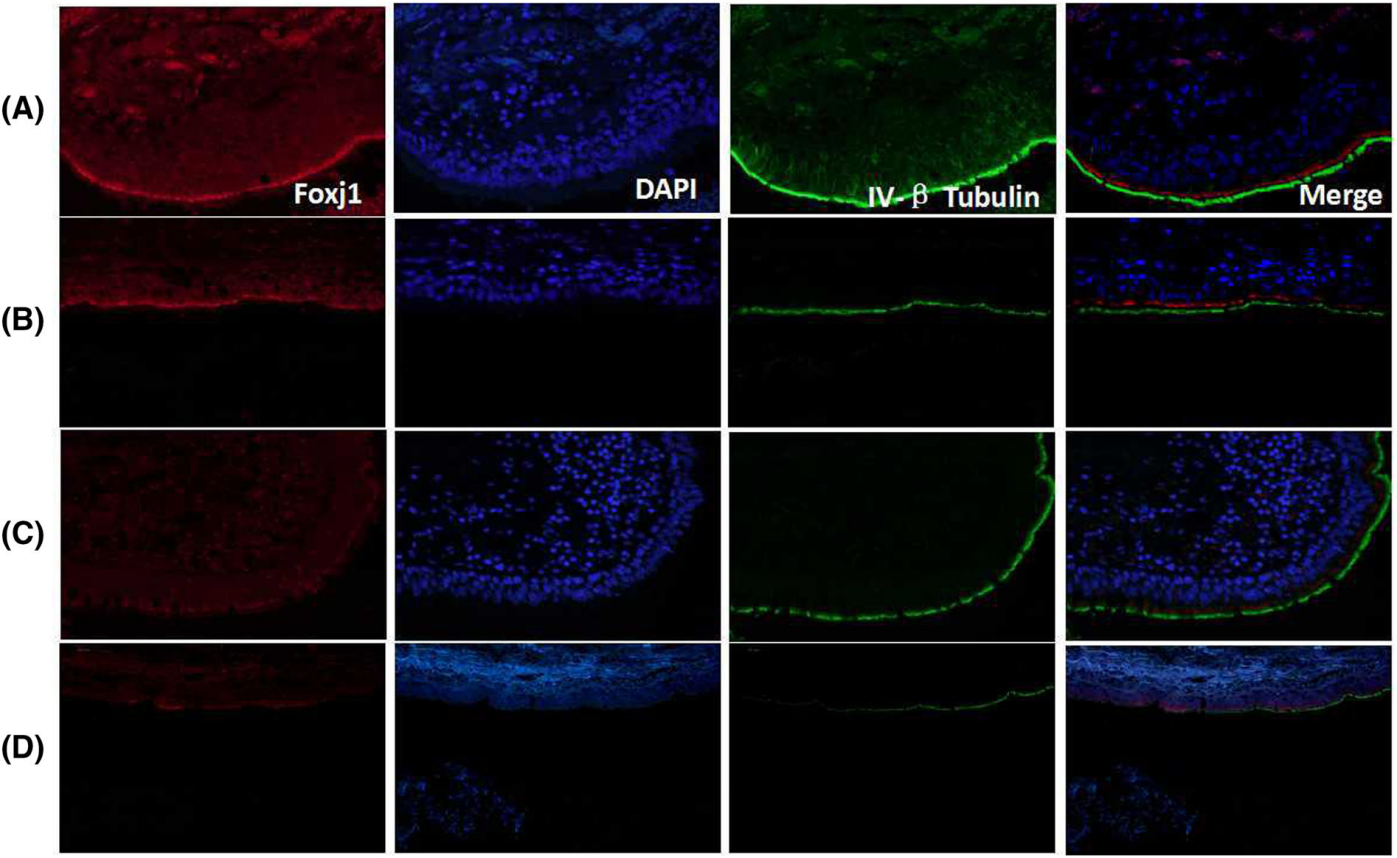
Expression of forkhead box protein j1 (Foxj1) by immunofluorescence (IF) staining in airway epithelium of normal control and non‐cystic fibrosis bronchiectasis (non‐CF BE) patients with different Bronchiectasis Severity Index (BSI) scores (×400 magnification; A, normal control; B, non‐CF BE mild group; C, non‐CF BE moderate group; D, non‐CF BE severe group).

Expression levels of Foxj1 were measured with IF staining. Compared with the clearly showed thicker and more nebulous pattern in the cilia of control subjects, Foxj1 staining of airway epithelium from non‐CF BE patients was thinner and less bright, with weaker fluorescence intensity and fewer positive area. The expression level of Foxj1 analysed by Image J was (2.69 ± 1.09) × 10^6^ significantly lower than that in the control group (6.67 ± 1.15) × 10^6^ (Figures 4 and 5, *P* = 0.001).

**FIGURE 6:**
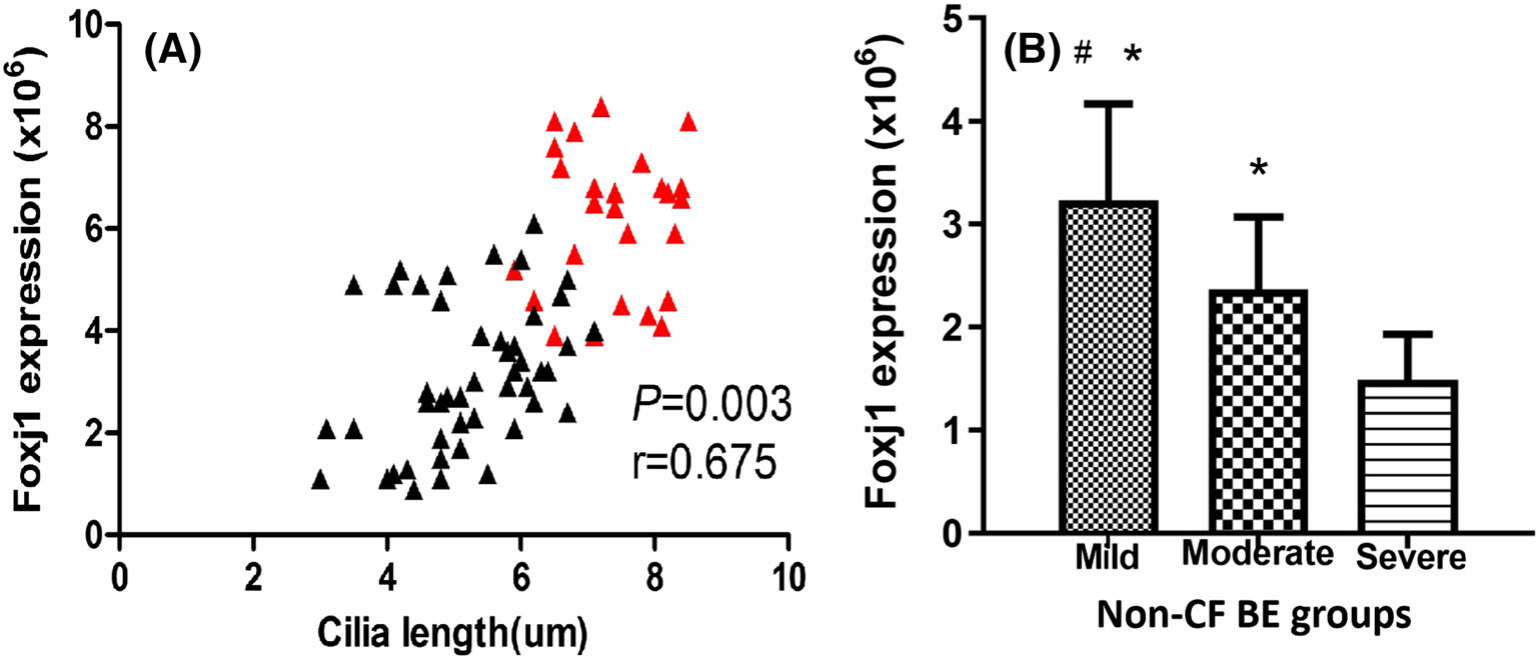
(A) Correlation between cilia length and forkhead box protein j1 (Foxj1) level (*P* = 0.003, r = 0.675; black points, non‐cystic fibrosis bronchiectasis [non‐CF BE] group; red points, control group). (B) Comparison of Foxj1 expression in non‐CF BE groups with different bronchiectasis severity index (BSI) scores (#*P <* 0.05 versus moderate group, *P < 0.05 versus severe group).

To further understand the relationship between Foxj1 level and cilia length, correlation analysis was performed. The result indicated a positive correlation between cilia length and Foxj1 level in patients with non‐CF BE (Figure 6, *P* = 0.003, r = 0.675). We further compared the Foxj1 expression levels among non‐CF BE patients with different BSI scores. The results showed that the level of Foxj1 expression was highest in patients from mild group (3.23 ± 0.94) × 10^6^ and declined in the moderate group (2.37 ± 0.70) × 10^6^. Patients in severe group expressed the lowest level of Foxj1 (1.49 ± 0.44) × 10^6^ compared with the other two groups (*P* = 0.001, F = 18.484, shown in Figure 6), suggesting that Foxj1 expression level might be associated with disease severity of non‐CF BE. Patients with declined Foxj1 expression seemed to be more severe.

## 5 DISCUSSION

The present study compared the airway histopathological condition of non‐CF BE patients and control subjects. Our results demonstrated cilia disorder (mainly, shortening of cilia or absence of cilia architecture) in non‐CF BE patients, accompanied with decline of Foxj1 expression in the airway epithelium. Further analysis indicated that Foxj1 level was positively correlated to cilia length, but negatively correlated to disease severity. Our results suggested that downregulation of Foxj1 might play a key role in the pathogenesis and development of non‐CF BE by modulating airway cilia function. This is important to better understand the pathogenesis of the disease.

To our knowledge, the pathogenesis of non‐CF BE is multifactoral and not clearly understood yet.^27^ Cilia had been demonstrated to play an important role in epithelial morphogenesis. The coordinated beating and length of motile cilia are crucial for mucociliary clearance process. If the cilia are shorter than the normal average of 6 to 7 mm, the mucus gel layer cannot be propelled in a normal fashion.^19^, ^28^ Thus, cilia loss, decreased ciliated cell numbers and shortened cilia likely all contribute to the occurrence of non‐CF BE. In the present study, we found multiple inflammatory cell infiltrations (including eosinophilic, lymphocytic and neutrophilic cell inflammation) in the airway of non‐CF BE patients, as well as epithelial and goblet cell hyperplasia and hypertrophy. It demonstrated that the airway epithelium of non‐CF BE was in chronic inflammation status, and was consistent with the results of previous studies.^9^, ^24^, ^27^ The result of IHC in our study indicated that patients with non‐CF BE also showed cilia impairment in the airway epithelium, mainly manifested as untidy and irregular cilia structures with shortened cilia or absence of cilia architecture. The median cilia length in non‐CF BE patients was shorter than that in the control group, (5.34 ± 0.89) μm versus (7.34 ± 0.71) μm, respectively. This was also in accordance with the findings in the previous researches, suggesting that cilia dysfunction promoted the development of non‐CF BE. Nevertheless, the mechanism to initiate the process of airway epithelial cilia disorder is still unclear up to date.

To initiate centriolar duplication and increase expression of the key components of cilia, the fox family has been under investigation in recent years. Genetic studies have identified Foxj1 as a key factor in modulating differentiation of motile ciliated cell types and ciliary motility^12^ by controlling cilia‐related genes expression and cooperate with other transcription factors to enhance specific populations of ciliation in different tissues.^29^ Suppressed Foxj1 expression has led to shorter cilia length in cilia cell cultures, whereas overexpression of exogenous Foxj1 reversed the inhibition of cilia growth. Foxj1 knockout mice had left–right asymmetry and airway defects.^15^

However, there has been lack of relative studies illustrating whether Foxj1 initiated the pathogenesis of non‐CF BE by modulating airway cilia dysfunction. In the current study, we found that the expression of Foxj1 declined in the airway epithelium of non‐CF BE, compared with control subjects. The level of Foxj1 was positively correlated to the airway cilia length, as patients with lower Foxj1 expression showed shorter cilia length, indicating that Foxj1 participated in the development of non‐CF BE by regulating airway cilia growth. Moreover, our results showed that the expression of Foxj1 differed among patients with different disease severity. Patients with less Foxj1 expression got higher BSI scores, suggesting that Foxj1 may also play a key role in the process of disease development by mediating severity of non‐CF BE.

## 6 CONCLUSION

The current study indicated that downregulation of Foxj1 might play an important role in the pathogenesis and development of non‐CF BE by modulating airway cilia function. Our result may supply theory evidence for better explaining the mechanism to initiating the pathogenesis of non‐CF BE. Nevertheless, further researches based on animal experiments are needed for clarifying the exact mechanism to modulate Foxj1 expression on airway epithelium and how Foxj1 mediate cilia disorder in non‐CF BE.

## AUTHOR CONTRIBUTIONS

Conceptualization: Xiao‐Ling Zou, Hai‐Ling Yang, Yu‐Qi Zhou and Tian‐Tuo Zhang. Data curation: Xiao‐Ling Zou, Hai‐Ling Yang, Wen‐Wen Ding and Hai‐Ke Li. Formal analysis and investigation: Xiao‐Ling Zou and Hai‐Ling Yang. Methodology: Xiao‐Ling Zou, Hai‐Ling Yang, Wen‐Wen Ding and Hai‐Ke Li. Project administration: Yu‐Qi Zhou and Tian‐Tuo Zhang. Software: Xiao‐Ling Zou and Hai‐Ling Yang. Supervision: Yu‐Qi Zhou and Tian‐Tuo Zhang. Roles/Writing ‐ original draft: Xiao‐Ling Zou and Hai‐Ling Yang. Writing ‐ review & editing: Yu‐Qi Zhou and Tian‐Tuo Zhang.

## CONFLICT OF INTEREST STATEMENT

There were no conflicts of interest, and there was no funding for this article.

## ETHICS STATEMENT

The study conformed to the guidelines of the amended Declaration of Helsinki and has obtained the approval from the Institutional Review Boards of The Third Affiliated Hospitals of Sun Yat‐sen University [No. (2018)02‐158‐01]. All patients have given their written consents.

## Data Availability

Research data are not shared.
